# Epigenetic silencing is a barrier to editing the X chromosome using the SEC-based CRISPR/Cas9 knock-in protocol in *C. elegans*

**DOI:** 10.17912/micropub.biology.001974

**Published:** 2026-01-21

**Authors:** Ryka Iyer, Simon Ferreria, Laahya Guvvala, Hugh Borden, Aariv Arora, Rebecca Shirsat, Ryan Doonan

**Affiliations:** 1 High School CRISPR Academy, Austin, Texas, United States; 2 Stuart Country Day School, Princeton, New Jersey, United States; 3 Livingston High School, Livingston, New Jersey, United States; 4 Downingtown S.T.E.M. Academy, Downingtown, Pennsylvania, United States; 5 Mercer Island High School, Mercer Island, Washington, United States; 6 Stargate School, Thornton, Colorado, United States; 7 Austin Community College, Austin, Texas, United States; 8 Eureka Program, Biology and Chemistry Division, New York, New York, United States

## Abstract

Self-excising-cassette (SEC)-based CRISPR/Cas9 knock-in is widely used for generating endogenous fluorescent protein tags in *C. elegans* . Here, we report a lack of success targeting the X chromosome using this method. CRISPR/Cas9 works as intended, but subsequent floxing of the SEC is blocked. Given that the X chromosome is epigenetically silenced in primordial germ cells (PGCs), this is a logical result. To circumvent this barrier, we suppressed polycomb repressive complex 2 (PRC2) with RNAi to transiently and reversibly reduce silencing in the PGCs, creating a brief window where the X chromosome is amenable to floxing without compromising germ line development. Overall, our results reveal a previously unrecognized limitation of SEC-based CRISPR/Cas9 knock-in and identify a reliable workaround for tagging proteins encoded on the X chromosome.

**Figure 1. Epigenetic silencing is a barrier to editing the X chromosome using the SEC-based CRISPR/Cas9 knock-in protocol in C. elegans:**
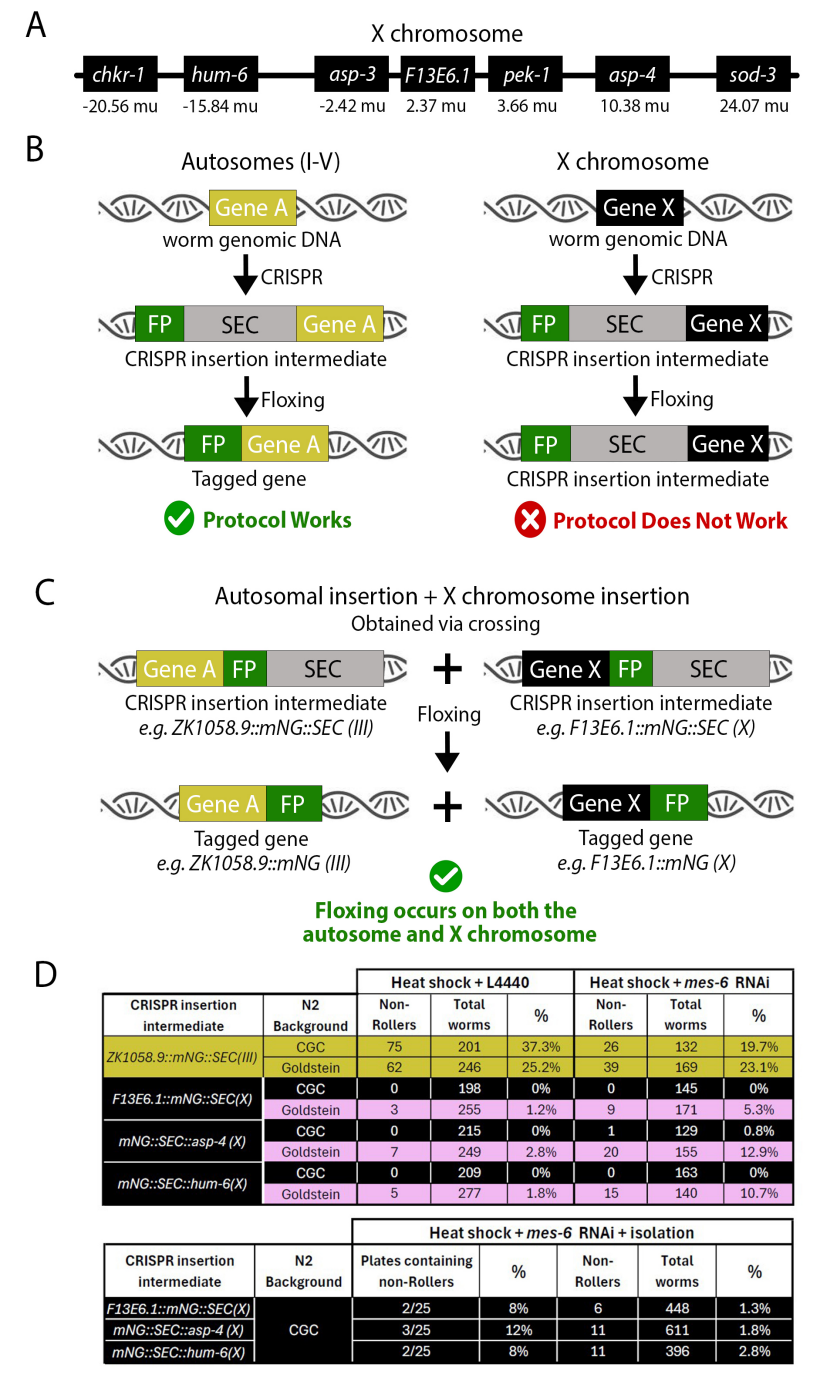
*(A)* Representative diagram of the X chromosome showing the relative position and map unit coordinates of the seven genes targeted for fluorescent protein (FP) knock-in in this study.&nbsp; Note that these genes are in very different regions of the X chromosome from -20.56 mu to +24.07 mu. *(B)* SEC-based CRISPR knock-in works as intended on autosomes, but not the X chromosome. Autosomal genes are represented on the left in yellow as “Gene A”, whereas X chromosome genes are represented on the right in black as “Gene X”. Note that in both cases it is possible to create the CRISPR insertion intermediate, but the self-excising cassette (SEC) cannot be autonomously floxed from the X chromosome. In other words, heat shock does not lead to inducible Cre expression if the SEC is inserted on the X chromosome. *(C)* A workaround for the problem in Fig. 1B is to cross the X chromosome CRISPR insertion intermediate into an autosomal CRISPR insertion intermediate genetic background. Heat shock results in floxing of the SEC from both the autosome and X chromosome, presumably via expression of Cre from the autosomal SEC. *(D)* Top table: Goldstein N2 stock is weakly permissive to X chromosome floxing and *mes-6* RNAi treatment enhances this permissiveness fivefold (see pink rows). For comparison, a representative example of autosomal floxing efficiency is shown in the yellow rows. Bottom table: It is possible to achieve X chromosome floxing in CGC N2 stock, but only if *mes-6* RNAi is used and screen sensitivity is increased.

## Description

Self-excising-cassette (SEC)-based CRISPR/Cas9 knock-in is a powerful and scalable system for creating endogenous fluorescent protein tags in *C. elegans* (Dickinson et al. 2015). Indeed, we have successfully tagged over 50 proteins using this approach (DeMott et al. 2021, Huang et al. 2021, McDonald et al. 2023, Witten et al. 2023). However, we were unsuccessful in our attempts to tag proteins encoded on the X chromosome (0/7 or 0% success, Fig. 1A and Fig. 1B ). Interestingly, we had no issues creating the CRISPR insertion intermediate on the X chromosome, meaning X chromosome DNA is accessible and amenable to CRISPR/Cas9 targeting and homology directed repair (HDR) ( Fig. 1B ). However, we were not able to induce self-excision of the SEC from the CRISPR insertion intermediate via the heat-shock-inducible Cre/lox system (i.e. floxing) on the X chromosome. Indeed, the entire X chromosome appears to be affected, as determined by systematic targeting from end to end ( Fig. 1A ). We have not encountered this problem when tagging proteins encoded on autosomes ( Fig. 1B, n=53 unique gene targets, see Methods for more details).&nbsp;

The X chromosome in *C. elegans* differs from autosomes in structure, gene content, regulation, chromatin state, and developmental roles (Strome et al. 2014). For example, the X chromosome is epigenetically silenced as facultative heterochromatin within the primordial germ cells (PGCs, i.e. Z2 and Z3) (Kelly et al. 2002). This is the result of repressive H3K27me3 histone methylation mediated by MES proteins of polycomb repressive complex 2 (PRC2) (Kelly et al. 2002, Fong et al. 2002, Strome and Updike 2015). These epigenetic marks distinguish the X chromosome from autosomes in Z2 and Z3 and this silencing is essential for maintaining germ line integrity and totipotency (Schaner and Kelly 2006, Seydoux and Braun 2006, Frøkjaer-Jensen et al. 2008, Shirayama et al. 2012, Gleason and Chen 2022). Given that floxing of the SEC from the CRISPR insertion intermediate targets Z2 and Z3 (it is carried out during L1) and requires heat-shock-induced transcription of Cre recombinase via the SEC, epigenetic silencing likely explains why the protocol does not work on the X chromosome ( Fig. 1B ).

To test this hypothesis, we first investigated whether we could circumvent this barrier by floxing worms at later stages of development (L2 to young adult). Presumably, this would target expanded populations of germ cells at various stages of the cell cycle and/or meiosis. Unfortunately, this did not result in floxing for any of the X-linked CRISPR insertion intermediates tested. We next investigated whether an autosomal CRISPR insertion intermediate is sufficient to promote floxing of the SEC from an X chromosome CRISPR insertion intermediate. To do this, we created dual CRISPR insertion intermediate strains via crossing ( Fig. 1C ). For example, we crossed *F13E6.1 ::mNeonGreen::SEC(X)* into a *ZK1058.9 ::mNeonGreen::SEC(III)* genetic background ( Fig. 1C ). We find that heat-shocking such dual CRISPR insertion intermediate strains during L1 results in floxing of the SEC from both the X chromosome and autosome ( Fig. 1C, see Methods for more details). Thus, the X chromosome is accessible and amenable to floxing if/when Cre recombinase is available. This supports our hypothesis that expression of Cre recombinase via the X chromosome is the barrier to floxing, rather than site-specific recombination. This is consistent with our ability to edit X chromosome DNA via CRISPR/Cas9 and homology-directed repair (HDR) in germ cells derived from Z2 and Z3 in the adult hermaphrodite gonad syncytium ( Fig. 1B ). Collectively, our analysis suggests that the chromatin state of the X chromosome allows genetic recombination, but probably not Cre recombinase transcription throughout germ line development.

Our results suggest that the SEC-based CRISPR/Cas9 knock-in protocol was destined to fail for the X chromosome. However, when searching the Caenorhabditis Genetics Center (CGC) database, we discovered that four different proteins encoded on the X chromosome had been successfully tagged using this protocol (Heppert et al. 2018). This discrepancy in outcomes could reflect genetic differences between CGC N2 stock and Goldstein laboratory N2 stock. Indeed, laboratory N2 stocks are known to have considerable genetic variability (Weber et al. 2010). To test this hypothesis, we first outcrossed our X chromosome CRISPR insertion intermediate strains to Goldstein N2 stock. This did not permit floxing of the SEC, suggesting that any relevant genetic differences between the Goldstein and CGC N2 stocks are likely complex rather than attributable to a single gene variant. Thus, we next repeated the SEC-based CRISPR/Cas9 knock-in protocol using Goldstein N2 stock to target three genes on the X chromosome, exactly as before with CGC N2 stock. Remarkably, all three genes were amenable to X chromosome floxing in the Goldstein N2 background, although floxed worms were rare (only ~2% of screened worms, versus ~25% for an autosomal gene) ( Fig. 1D, top table, pink versus yellow rows). This suggests that X chromosome silencing is weakly permissive in the Goldstein N2 stock, allowing recovery of rare floxed worms using the standard SEC-based CRISPR/Cas9 knock-in protocol (Heppert et al. 2018). This was interesting, but it does not solve the problem of using CGC N2 stock, which is available to all researchers worldwide.&nbsp;

Given that the molecular mechanisms of epigenetic silencing of the X chromosome are well understood, we next aimed to transiently relieve this silencing in Z2 and Z3 by reducing PRC2 activity via *mes-6* RNAi, thereby weakening H3K27me3-mediated repression. Prior studies using X-linked reporters have shown that such de-repression is both possible and reversible (Kelly et al. 2002, Bender et al. 2004, Patel et al. 2012). Our goal was to generate a brief window in which the X chromosome is sufficiently accessible to permit heat-shock–induced Cre expression and SEC excision without compromising Z2/Z3 identity or germ line development. This strategy yielded an average fivefold increase in floxed progeny in the Goldstein N2 background ( Fig. 1D, top table, pink rows). In contrast, the CGC N2 background appeared to remain entirely resistant to floxing ( Fig. 1D, top table, black rows).&nbsp;This suggests that the Goldstein N2 stock is more sensitive to X chromosome de-repression, consistent with silencing being weakly permissive in this genetic background.&nbsp;

Our results indicate that it is more difficult to recover X chromosome excisions versus autosomal excisions, even in a permissive background + *mes-6* RNAi ( Fig. 1D, top table). Thus, although screening pooled F _1_ progeny is generally sufficient for successful floxing (Dickinson et al. 2015), we reasoned that pooling may be obscuring very rare floxed worms in CGC N2 treated with *mes-6* RNAi. Indeed, isolating individual L1s following heat shock + *mes-6* RNAi revealed that 2–3 out of 25 L1 worms (i.e. 8-12%) produced floxed F _1_ progeny at very low frequency (1-3% non-Rollers) ( Fig. 1D, bottom table). Thus, we were able to find a straightforward workaround for floxing X chromosome CRISPR insertion intermediates in CGC N2 stock, which was our primary goal.&nbsp; &nbsp;

Overall, we have identified X chromosome epigenetic silencing in the PGCs as a previously unappreciated barrier to SEC-based CRISPR/Cas9 knock-in in *C. elegans* . Although the SEC integrates efficiently on the X chromosome, heat-shock-induced Cre expression may be restricted, preventing autonomous excision. We demonstrate that transient depletion of PRC2 using *mes-6* RNAi allows floxing, suggesting that this creates a reversible window of X-linked transcription of Cre. Although efficiency remains much lower than for autosomes, combining *mes-6* RNAi with individual worm isolation provides a reliable workaround for achieving X chromosome floxing in CGC N2 . These observations refine our understanding of germ line chromatin constraints in *C. elegans* and offer practical guidance for CRISPR/Cas9 editing on the X chromosome.

## Methods

**CRISPR/Cas9 knock-in**

In general, SEC-based CRISPR/Cas9 knock-in was done as previously described (Dickinson et al. 2015, Huang et al. 2021, DeMott et al. 2021). All modifications to this protocol unique to this study and/or the X chromosome are discussed below.

**X chromosome versus autosome genes** The X chromosome genes evaluated in this study were *asp-3* , *asp-4* , *chkr-1* , *F13E6.1* , *hum-6* , *pek-1* , and *sod-3* . For comparison, 53 genes were targeted across autosomes I–V (DeMott et al. 2021, Huang et al. 2021). The distribution of these genes by chromosome was as follows: I (7), II (11), III (14), IV (12), V (9). All were successfully targeted. The X chromosome genes targeted in the Heppert et al. 2018 study were *mes-1* , *dlg-1* , *klp-4* , and *klp-8* .

**CGC N2 versus Goldstein N2** CGC N2 stock was obtained from the Caenorhabditis Genetics Center in 2019. Goldstein N2 stock was obtained from the Glow Worms collection at The University of Texas at Austin in 2023, brought there by Daniel Dickinson from the University of North Carolina at Chapel Hill in 2017. Outcrossing experiments involved six rounds of serial backcrossing of CGC N2 CRISPR insertion intermediate strains into Goldstein N2 males. CRISPR/Cas9 knock-in was performed in CGC N2 and/or Goldstein N2 stocks (see strain details below).

**Timing variation for heat shock** To test whether germ cell developmental stage influences floxing efficiency, heat shock was performed on mixed-stage populations rather than L1 larvae. The goal was to target diploid germ cells descended from Z2 and Z3 (L2 to young adult), as well as haploid gametes (young adults). For the screen to be sensitive to haploid floxing and/or floxing in F _1_ heterozygotes, we screened both the F _1_ and F _2_ for non-Rollers.

**Dual intermediate crossing strategy** To test whether autosomal SEC insertions could rescue X chromosome floxing, four different X-linked CRISPR insertion intermediates were crossed into a *ZK1058.9 ::mNeonGreen::SEC(III)* genetic background (see RTD5, RTD7, RTD17, and RTD19 in Reagents). These dual CRISPR insertion intermediate strains were subjected to L1 heat shock as previously described (Dickinson et al. 2015). Floxing efficiency was not scored, but in all cases recovery of dual floxed animals was possible. Although this represents a workaround for X chromosome floxing, it is worth noting that these crosses were tedious (i.e. often unsuccessful, requiring repeating), given that they involve crossing Roller males into Roller hermaphrodites (the SEC produces a Roller phenotype via the *sqt-1 (d)* mutation).

**RNAi and heat shock**

The *mes-6* RNAi clone was verified by Sanger sequencing.&nbsp; We then confirmed that it could induce a maternal effect sterile phenotype in the F _1_ and F _2_ when fed to CGC N2 P _0_ hermaphrodites. For floxing experiments, 15-25 asynchronous L1 larvae were pooled on L4440 or *mes-6* RNAi plates for 4 hours at 20°C, moved to NGM plates for heat shock for 4 hours at 34°C, then returned to 20°C. Given that Z2 and Z3 are in G2 arrest during L1, this represents 30-50 PGCs, 60-100 X chromosomes, and 120-200 X chromatids exposed to heat shock and RNAi. As young adults, these worms were either: (1) split and pooled in groups of 5 on NGM plates and allowed to lay eggs for 3 hours, then removed; or (2) isolated on individual NGM plates and allowed to lay a full brood of eggs. Eggs were counted and the subsequent fraction of non-Roller F _1_ progeny was scored.&nbsp;&nbsp;

## Reagents

**Table:** 

**Strain**	**Genotype**	**Background**
GLW44	*ZK1058.9 ::mNG::SEC(III) *	CGC N2
GLW58	* mNG::SEC:: asp-3 (X) *	CGC N2
RTD5	*ZK1058.9 ::mNG::SEC(III); mNG::SEC:: asp-3 (X) *	CGC N2
RTD6	*ZK1058.9 ::mNG(III); mNG:: asp-3 (X) *	CGC N2
GLW94	*F13E6.1 ::mNG::SEC(X) *	CGC N2
RTD7	*ZK1058.9 ::mNG::SEC(III); F13E6.1 ::mNG::SEC(X) *	CGC N2
RTD8	*ZK1058.9 ::mNG(III); F13E6.1 ::mNG(X) *	CGC N2
RTD2	* mNG::SEC:: asp-4 (X) *	CGC N2
RTD17	*ZK1058.9 ::mNG::SEC(III); mNG::SEC:: asp-4 (X) *	CGC N2
RTD18	*ZK1058.9 ::mNG(III); mNG:: asp-4 (X) *	CGC N2
RTD4	* mNG::SEC:: hum-6 (X) *	CGC N2
RTD19	*ZK1058.9 ::mNG::SEC(III); mNG::SEC:: hum-6 (X) *	CGC N2
RTD21	*ZK1058.9 ::mNG(III); mNG:: hum-6 (X) *	CGC N2
RTD1	* mSc::SEC:: sod-3 (X) *	CGC N2
RTD3	* mNG::SEC:: pek-1 (X) *	CGC N2
RTD13	*mNG::SEC::chkr-1(X)*	CGC N2
RTD22	*ZK1058.9 ::mNG::SEC(III) *	Goldstein N2
RTD11	*F13E6.1 ::mNG::SEC(X) *	Goldstein N2
RTD24	* mNG::SEC:: asp-4 (X) *	Goldstein N2
RTD25	* mNG::SEC:: hum-6 (X) *	Goldstein N2
